# Extracting Foreign Bodies from the Stomach

**Published:** 1890-08

**Authors:** 


### EXTRACTING FOREIGN BODIES FROM THE STOMACH.

The removal of foreign bodies from the stomach by the so-called potato cure, consists in requiring the patient to eat large quantities of potatoes, which have the effect of proportionately dilating the whole intestinal canal, so that the foreign body is enveloped and cannot cling to any part during its passage. Dr. Salzer showed at the clinic of Professor Billroth, several foreign bodies which, in this way, had been removed—one of these being a weight of 5½ drachms, which had been swallowed by a child ; the second, a set of artificial teeth, upwards of 5 centimetres long, and 3 centimetres broad, and the third was a needle. Many gastrotomies could be obviated by this method in the case of swallowed foreign bodies.

